# Targeted inhibition of phosphatidyl inositol-3-kinase p110β, but not p110α, enhances apoptosis and sensitivity to paclitaxel in chemoresistant ovarian cancers

**DOI:** 10.1007/s10495-013-0807-9

**Published:** 2013-01-31

**Authors:** Ju-yeon Jeong, Kyung-Sul Kim, Ji-Sook Moon, Ji-ae Song, Sung-ho Choi, Kwang-il Kim, Tae-Heon Kim, Hee-Jung An

**Affiliations:** 1 Department of Pathology, College of Medicine, CHA University, 351 Yatap-dong, Gyeonggi-Do Seongnam Si Bundang-gu, 463-712 Republic of Korea; 2 Department of Biomedical Science, CHA University, Seoul, South Korea; 3 Institute for Clinical Research, CHA University, Sungnam, South Korea; 4 Isu Abxis Co., Ltd, Seoul, South Korea

**Keywords:** Ovarian cancer, Chemoresistance, Phosphatidyl inositol-3-kinase p110β, Small interfering RNA, Target therapy

## Abstract

The phosphatidylinositol 3-kinase (PI3K) pathway is one of the critical signaling cascades playing important roles in the chemoresistance of human cancer cells, including ovarian cancer. In this study, we investigated the potential of targeting the PI3K p110β-isoform as a novel approach to overcome the chemoresistance in ovarian cancer. The effects on apoptosis, cell viability, proliferation and migration in chemoresistant ovarian cancer cell were determined following targeted p110β inhibition by small interfering RNA (siRNA). Seven paclitaxel (PTX)-resistant sublines (SKpacs and A2780pac) were produced from SKOV3 and A2780 ovarian cancer cell lines. We, first, evaluated the expression of PI3K p110 isoforms in chemosensitive and chemoresistant ovarian cancer cell lines and patient specimens, and found that p110β-isoform was significantly overexpressed both in a panel of ovarian cancer samples, and in PTX-resistant sublines compared with their parent cell lines. RNA interference-mediated p110β silencing augmented PTX-mediated apoptosis (31.15 ± 13.88 %) and reduced cell viability (67 %) in PTX-resistant cells, whereas targeting p110α did not show a significant change in cell viability and apoptosis. In addition, p110β silencing impaired cell proliferation (60 %) in PTX-resistant SKpac cells. We also found the combined treatment group with p110β siRNA and PTX showed a significant inhibition of tumor growth of SKpac cells compared to the PTX-only treated group in a xenograft nude mouse model. Thus, the siRNA-mediated silencing of PI3K p110β resensitizes PTX-resistant ovarian cancer cells, and may be a useful therapeutic strategy for PTX-resistant ovarian cancers.

**Electronic supplementary material:**

The online version of this article (doi:10.1007/s10495-013-0807-9) contains supplementary material, which is available to authorized users.

## Introduction

Ovarian carcinoma has the highest mortality rate among the gynecological malignancies. At present, the surgical cytoreduction and systemic paclitaxel (PTX)-platinum combination chemotherapy are the choice of treatment. However, more than 70 % of patients with advanced stage develop the recurrence within 5 years [1]. The major reason of the failure of primary treatment is the development of chemoresistance [2]. PTX is an antimicrotubule agent that is currently used as a first-line chemotherapy for the treatment of ovarian, breast, and non-small cell lung cancers [3]. Therefore, the control of drug-resistance against PTX is one of the important issues in the improved treatment of ovarian cancer. The cause of chemoresistance is multifactorial, and PTX resistance can be mediated by the overexpression of P-glycoprotein and altered expression or post-translational modification of β-tubulin or other microtubule regulatory proteins [4]. Still, the molecular mechanisms of chemoresistance to PTX remain to be investigated.

The phosphatidylinositol 3-kinase (PI3K)/AKT pathway is one of the critical signaling cascades that are important in the chemoresistance of human cancer cells [5, 6]. The PI3K signaling pathway is involved in cellular processes such as cell survival, proliferation and apoptosis, and in many physiologic processes [7, 8]. PI3Ks are lipid kinases that consist of three different classes (class I, II, and III) according to their structures and mechanism of activation. Class I PI3Ks are the best-characterized enzymes, including class IA (p110α, p110β, and p110δ) and class IB (p110γ). They are composed of a catalytic subunit (p110) and a regulatory subunit (p85/p55/p101). Aberrant activation of this pathway has been widely-reported in many human cancers, including ovarian cancer [9, 10]. Amplification and/or mutations of the PIK3CA gene encoding the p110α catalytic subunit of PI3K have been reported in many ovarian cancers [11, 12]. Furthermore, recent studies have reported that inhibition of the PI3K/AKT pathway increases the efficacy of chemotherapeutic agents in human malignancies including ovarian cancers [13, 14]. A previous study showed that overexpression of PI3K in ovarian cancer cells resisted to PTX and that inhibition of PI3K activity increased sensitivity to PTX on ovarian cancer cells [15]. The most widely-used approach to inhibit the PI3K/AKT pathway is to target PI3K itself, such as by the use of Wortmannin and LY294002. However, these inhibitors have a limitation on in vivo use due to their toxic effect of normal cells, because the PI3K pathway is involved in insulin signaling and immune reaction. Therefore, targeting the specific isoform of PI3K has recently been tried to reduce cancer cell growth and survival without side effects [16, 17] .

In the present study, we evaluated the expression of PI3K p110 isoforms in human ovarian carcinoma tissue samples and cell lines, and found that β-isoform was significantly overexpressed in ovarian carcinoma tissues and chemoresistant ovarian cancer cell lines. We, therefore, investigated whether the specific targeting to PI3K p110β could impair the cell growth and survival of chemoresistant ovarian cancer cells in vitro and in vivo. Our results demonstrate for the first time that PI3K p110β-isoform is overexpressed in a subset of ovarian carcinoma samples, and that the selective inhibition of PI3K p110β sensitizes chemoresistant ovarian cancer cells to PTX.

## Materials and methods

### Tumor tissue samples and cell lines

Fresh tissue samples of 40 ovarian epithelial tumors, including five benign and 35 serous type malignant tumors, were obtained at the time of surgery from patients who had undergone oophorectomies for ovarian epithelial tumor at the CHA Bundang Medical Center. Samples were immediately frozen in liquid nitrogen and stored at −80 °C. Using frozen sections, it was confirmed that the purity of tumor cells was nearly 80 % of tissue. Western blotting for the α, β, γ, and δ isoforms of PI3K p110 was performed to assess which isoforms of PI3K p110 was more obviously overexpressed in ovarian carcinomas compared to benign tumors. This study was approved by the Ethical Committee of the CHA Bundang Medical Center. Informed consent was obtained from each patient prior to surgery.

The SKOV3 and A2780 human ovarian carcinoma cell lines were obtained from American Type Culture Collection (Manassas, VA, USA). PTX-resistant sublines (SKpac and A2780pac) were produced from parent cell line, SKOV3 and A2780, respectively, by continuous exposure of cells to a stepwise escalating concentration of PTX over 8 months.

These cell lines were maintained in MaCoy’s 5A medium (Gibco Invitrogen, Carlsbad, CA, USA) with 10 % fetal bovine serum, penicillin 100 IU/ml, and streptomycin 50 μm/ml in a humidified atmosphere containing 5 % CO_2_ at 37 °C. Cell viability was determined by the MTT assay as previously described [18].

### Transfection with PI3K p110α and β siRNA

PI3K p110α and p110β siRNAs were synthesized by Qiagen (Valencia, CA, USA). The day before transfection, 1 × 10^5^ cells were seeded into each well of a six-well plate. The next day, cells were transfected with annealed siRNA oligos (supplementary data Table S1) using Lipofectamine 2000 (Invitrogen, Carlsbad, CA, USA) according to the manufacturer’s instructions. At 24, 48, and 72 h after transfection, cells were harvested and prepared for subsequent study.

### Western blot

Cells were lysed in RIPA buffer (Biotech, Seoul, Korea) and centrifuged at 12,000 rpm at 4 °C for 20 min. Western blotting was performed as previously described [18]. Primary antibodies were used as follows; β actin 1:10,000, PI3Kβ 1:1,000, phospho serine 478 AKT 1:1,000, phospho threonine 308-AKT 1:500, mTOR 1:1,000, S6 ribosomal protein 1:1,000 (Cell Signaling Technologies, Danvers, MA, USA), phospho-mTOR 1:500 (Millipore, Billerica, MA, USA), bcl-2α 1:1,000 (Lab Vision. Fremont, CA, USA), DNA-PK 1:1,000 (Epitomics, California, USA), cyclin D1 1:1,000, E2F1 1:1,000 (Santa Cruz Biotechnology, Santa Cruz, CA, USA), cyclin E 1:1,000 (Upstate Biotechnology, Lake Placid, NY, USA), p27^kip1^ 1:500, p21^WAF1^/_Cip1_ 1:1,000, retinoblastoma (pRb) 1:1,000, NF-κB p65 1:1,000 (Neomarkers, Fremont, CA, USA), SKP2 1:500 (Zymed, South San Francisco, CA, USA), and LTA 1:500 (Abnova, Jhouzih St., Taipei, Taiwan).

### TUNEL assay

Cells (2 × 10^7^) were fixed with 75 % ethanol for 2 h at −20 °C. Apoptotic cells were analyzed using the In Situ Cell Death Detection kit (Roche, Mannheim, Germany) and detected by fluorescence activated cell sorting (FACS) (Becton–Dickinson, Franklin Lakes, NJ, USA) as previously described [18].

### Colony-forming assay

Cells were seeded at a density of 1 × 10^5^ cells per well in six-well plates. The next day, cells were transfected with siRNA and incubated for 48 h. Transfected cells were then replated at 300 cells per well in a gelatin-coated six-well culture dish. After 14 days, colonies were visualized using hematoxylin after fixation with 4 % paraformaldehyde for 10 min and then counted. Groups of >50 cells were scored as colonies.

### RT2 profiler™ PCR array

To analyze the apoptosis-related genes regulated by the p110β isoform, PCR array was done after p110β siRNA transfection. For first-strand cDNA synthesis, 1 µg of total RNA was reverse transcribed in a final reaction mix of 20 µl using a RT2 First Strand Kit (SuperArray Bioscience, Frederick, MD, USA) according to the manufacturer’s instructions. The qRT-PCR was performed with CFX96 (Bio-Rad) and universal cycling conditions (10 min at 95 °C, 15 s at 95 °C, 1 min 60 °C for 40 cycles) were carried out. Fold change in gene expression for all the genes was calculated using the comparative cycle Ct(∆∆Ct) method. The statistical calculation was based on the web-based program of RT2Profiler™ PCR Array Data Analysis (SuperArray Bioscience).

### Plasmid and transfection

The pcDNA3 BCL2 plasmid was purchased from Addgene (Cambridge, MA, USA). Cells of 1 × 10^5^ were seeded into each well of a six-well plate. The next day, cells were transfected with pcDNA3 BCL2 plasmid and annealed p110β siRNA oligos using Lipofectamine 2000 (Invitrogen, Carlsbad, CA, USA) according to the manufacturer’s instructions. At 48 h after transfection, cells were harvested and prepared for TUNEL assay. The efficiency of transfection of BCL2 plasmid was confirmed by Western blotting.

### Xenograft in vivo tumor model

All animal experiment protocols were approved by the Institutional Animal Care and Use Committee of CHA University. Five-week-old female BALB/c-nu Slc nude mice purchased from Central Lab. Animal Inc. (Seoul, Korea) were housed in a pathogen-free room. Mice received a subcutaneous implantation of the cells using 0.1 ml (3 × 10^7^cells/ml) per inoculation under the left shoulder (SKOV3) and right shoulder (SKpac). When the subcutaneous tumors were approximately 30 mm^3^ in size, mice were then assigned randomly to one of four groups (*n* = 6 per group) and treatment was initiated 20 days after the transplantation and carried out for 3 weeks as follows: Group 1, intraperitoneal (i.p.) PBS weekly and intratumoral PBS every 3 days; group 2, i.p. PTX weekly (25 mg/kg per injection) and, intratumoral PBS every 3 days; group 3, i.p. PTX weekly and intratumoral negative siRNA (1.2 nM) every 3 days; and group 4, i.p. PTX weekly and intratumoral PI3K p110β siRNA (1.2 nM) every 3 days.

Tumor growth was measured twice a week using a caliper and tumor volume was calculated using the following formula:

$$ {\text{Tumor volume }}\left( {a\, \times \, b_{ 2} } \right)/ 2 $$

where *a* is the widest diameter of the tumor and *b* is the diameter perpendicular to *a*. Animals were sacrificed after 55 days from the first day of treatment and the tumors were excised and weighed.

### Statistical analysis

The significant differences between groups were determined using a Student’s *t* test. A *p* value <0.05 was considered statistically significant. Statistical analysis was performed using the SAS statistics software package (SAS Enterprise Guide 4.1; SAS Institute, Cary, NC, USA).

## Results

### Production of chemoresistant sublines

Seven different sublines (SKpac-8, 11, 12, 13, 16, 17, and A2780pac) were generated. The IC50 levels for the SKOV3 versus Skpac cells and A2780 versus A2780pac were 22 nM: 7.8 μM and 5.4 nM: 430 nM, respectively (supplementary data, Table S2-5). This resistance paralleled the reduction of PTX-induced apoptosis in chemoresistant cells relative to their parental cells. Treatment of parental SKOV3 cell with 80 nM PTX for 48 h resulted in significant induction of apoptosis (98.24 %), whereas a marked reduction in apoptosis was seen in the PTX-resistant SKpac cells (1.1 %) with the same condition of PTX treatment (supplementary Fig. S1).

### PI3K p110β isoform is upregulated in ovarian cancer tissue and chemoresistant cancer cell lines

**Fig. 1:**
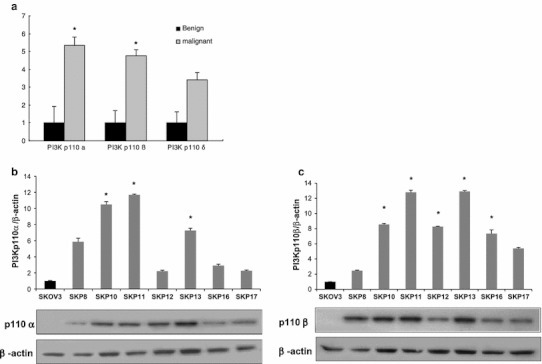
Protein expression of various isoforms of PI3K p110 by Western blotting in ovarian cancer tissues and cells. **a** The graph represents the intensity ratio of protein expression band of various isoforms of PI3K p110 in ovarian serous carcinoma tissues relative to serous benign tumors of each protein normalized to corresponding β-actin band. The relative folds of expression of PI3K p110α, β, and δ isoforms were 5.3-, 4.8-, and 3.4-fold, respectively, compared to the mean value of benign tumor tissues (* *p* < 0.05, *t* test). **b**, **c** Protein expression of p110α (B) and p110β (C) in chemoresistant SKpac cells were compared with parent SKOV3 cells. Representative experiment repeated twice with similar results. The bands were quantified by densitometric analysis. The intensity ratio to corresponding β-actin band was calculated. Columns denote relative-fold normalized to SKOV3 level

We performed Western blotting for various isoforms of PI3K p110 in 35 primary serous type ovarian cancer and 5 benign tumor samples to investigate which isoform was significantly overexpressed in this subset of ovarian cancer. The p110α and β isoforms showed statistically significant overexpression in ovarian cancer tissue compared to the benign tumor tissues (*p* < 0.05, Student’s *t* test). The relative folds of expression bands of PI3K p110α, β, and δ isoforms were 5.3-, 4.8-, and 3.4-fold, respectively, compared to the mean value of benign tumor tissues (Fig. 1a). However, the alteration of the p110δ was not statistically significant. The PI3K p110γ was not detected in the ovarian tumors. Intriguingly, the expression of PI3K p110β was significantly increased by 2.5–3.5-fold in the chemoresistant sublines compared to the parental cell line (*p* < 0.05, *t* test), whereas the increase of p110α was 1.5–3-fold, and was not statistically significant (Fig. 1b, c). Together, these results suggest that acquired chemoresistance is associated with increased expression of the p110β isoform rather than other isoforms of PI3K. We, therefore, selected the p110β-isoform for further study of chemoresistance by siRNA-mediated knockdown.

### Suppression of PI3K p110β overexpression by siRNA leads to downregulation of downstream targets of PI3K/Akt/mTOR pathway and alteration of cell cycle factors

**Fig. 2:**
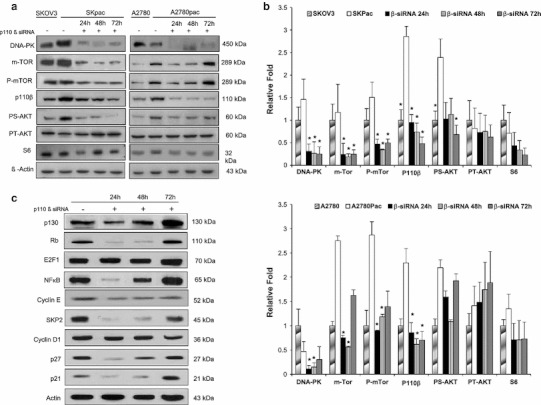
The expression of downstream targets and cell cycle-related proteins after PI3K p110β siRNA transfection. **a** The alteration of protein expression of downstream targets of PI3K pathway before and after PI3K p110β siRNA treatment in chemoresistant SKpac and A2780pac cells. The downstream effectors, pS-AKT(S478), DNA-PK, mTOR, and p-mTOR, as well as PI3K p110β were overexpressed in chemoresistant SKpac and A2780 pac cells. Those proteins were down-regulated after PI3K p110β siRNA treatment. However, pT-AKT (T308) was not altered in chemoresistant cells. **b** The graph represents the relative amount of each protein compared to their parent cells. Protein bands of (**a**) were quantitated by densitometric analysis. The intensity ratio to corresponding β-actin band was calculated. Representative experiment repeated twice with similar results (* *p* < 0.05, *t* test). **c** The effect of p110β knock-down on cell cycle regulatory proteins. SKOV3 cells were transfected with PI3K p110β siRNA for 24, 48, and 72 h and analyzed by western blotting with antibodies against different cell cycle-associated proteins. The amount of total actin serves as a loading control. The expression of pRB, p130, cyclin E, SKP2, and NFκB were obviously decreased by p110β knock-down, whereas cyclin D1 and E2F1 were not. Representative experiment repeated three times

The protein levels of phospho-Akt Ser 473, mTOR, phophorylated mTOR, DNA-PK, and S6 ribosomal protein were elevated along with p110β in chemoresistant SKpac and A2780pac cells, which were markedly decreased after p110β siRNA treatment (Fig. 2a, b). In contrast, there was no alteration in the levels of phospho-Akt Thr 308. These findings strongly indicate that p110β is the critical PI3K isoform driving PI3K pathway activation in this subset of cancer cells.

We then investigated the alteration of various cell cycle regulatory proteins induced by p110β siRNA treatment to assess the PI3K p110β-specific underlying mechanisms associated with tumor cell proliferation. Treatment of SKOV3 cells with p110β siRNA decreased the levels of p130, pRb, NFκB, cyclin E, and SKP2 at 24–48 h after transfection and recovered the expressions at 72 h, representing the transient knock-down effect of p110β siRNA (Fig. 2c). However, the level of cyclin D1 and E2F1 did not alter after p110β siRNA treatment. Unexpectedly, p27 and p21 were also decreased after p110β siRNA treatment.

### Specific inhibition of PI3K p110β but not p110α sensitizes chemoresistant SKpac cells to PTX

**Fig. 3:**
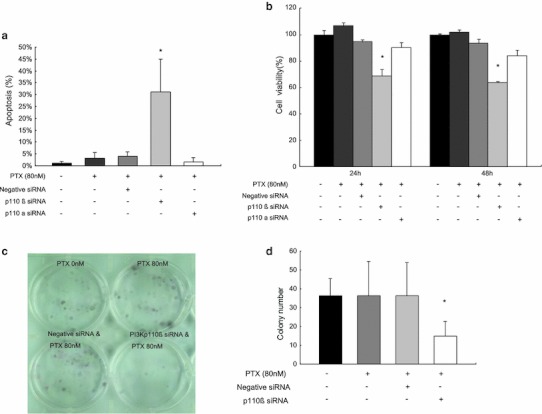
The effect of PI3K p110β siRNA on PTX-resistant SKpac cells in apoptosis and cell viability. **a** The graph represents the mean apoptotic cell rates after various treatments. Combined treatment of PI3K p110β siRNA and PTX resulted in markedly increased apoptosis (31.15 ± 13.88 %) in SKpac cells, whereas high-dose PTX alone induced low apoptosis (2.43 %). Control siRNA and p110α siRNA did not enhance apoptosis in combination with PTX (1.94 %). Representative experiment repeated three times (* *p* < 0.0001, *t* test). **b** MTT cell viability test of SKpac cells at 24 and 48 h after negative siRNA, PI3K p110β siRNA, and p110α siRNA treatment combined with high dose PTX. The cell viability at 24 h was 67, 90 %, respectively with PI3K p110β siRNA and p110α siRNA treatment, whereas it was 95 % with negative siRNA treatment. At 48 h, cell viability was 63, 84, and 94 %, respectively, with PI3K p110β siRNA, p110α siRNA, and negative siRNA treatment. Representative experiment repeated three times (* *p* < 0.05, *t* test). **c** Colony forming assay. SKpac cells after various treatment with PI3K p110β siRNA and PTX were seeded at 300 cells/well. Colonies were counted after 14 days. The experiment was repeated in triplicate. **d** The graph represents the mean numbers of colonies of cells with various treatment. The mean number of colonies formed in PTX-only treatment and in cells treated with PI3K p110β siRNA and PTX were 36.5 ± 18.1 and 14.8 ± 8.0, respectively. Representative experiment repeated three times (* *p* < 0.05, *t* test)

We examined the effects of combined treatment with p110β siRNA treatment (50 mmol/l) and PTX (80 nM) on apoptosis in chemoresistant SKpac cells by flow cytometry. The efficiency of PI3K p110β knockdown after siRNA transfection was confirmed by RT-PCR and Western blotting (supplementary Figs. S2a, b). We also examined the effect of p110α siRNA to assess whether p110α-specific inhibition might enhance apoptosis in these SKpac cells, because the p110α isoform was also increased in chemoresistant cells. The combined treatment of p110β siRNA and PTX resulted in a markedly increased apoptosis (31.15 ± 13.88 %) in SKpac cells (*p* < 0.0001, *t* test), whereas high-dose PTX alone induced low apoptosis (2.43 %). On the other hand, control siRNA or p110α siRNA did not enhance apoptosis in combination with PTX (1.94 %) (Fig. 3a). PI3K p110β siRNA treatment in SKpac cells reduced the cell viability by 67 and 63 % at 24 h and 48 h after siRNA treatment, respectively (*p* < 0.05, *t* test), whereas cell viability was not significantly changed when treated only with PTX, negative siRNA and p110α siRNA (Fig. 3b). These findings suggest that PI3K p110β silencing, but not p110α, sensitizes to PTX in PTX-resistant SKpac cells.

We also evaluated the effect of p110β siRNA on cell proliferation of the chemoresistant ovarian cancer cells under the condition of 80 nM PTX treatment. The transfection of p110β siRNA led to a 60 % decrease in colony numbers formed by SKpac cells, compared to the cells with PTX only and negative siRNA treatment (14.8 ± 8.0 vs. 36.5 ± 18.1 vs. 36.0 ± 17.5, *p* < 0.05, *t* test, Fig. 3c, d).

### PI3K p110β silencing impairs chemoresistant ovarian cancer tumor growth in vivo

**Fig. 4:**
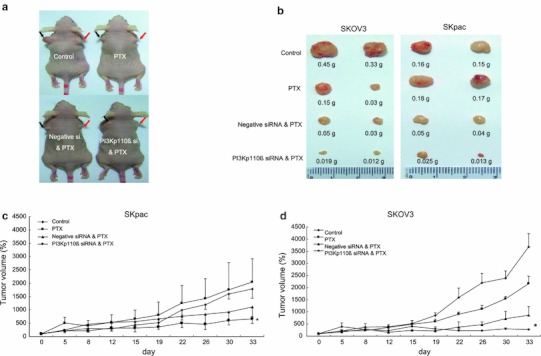
In vivo effect of PI3K p110β siRNA transfection on the growth of SKOV3 or SKpac xenografts in nude mice. Nude mice were inoculated s.c. with SKOV3 or SKpac cells. When the tumors reached an average size of approximately 30 mm^3^, mice were treated with PTX or siRNA for 3 weeks. **a** Representative results of the mice with SKOV3 (*left*) and SKpac (*right*) xenografts treated with PTX only or PTX and negative, or PTX and p110β siRNA. **b** Examples of tumor size and weight at the end of experiment of each group. The p110β siRNA + PTX treatment groups in SKpac xenografts was obviously suppressed as compared to the control and PTX-only treated groups. **c**, **d** Graphs depict tumor volumes for each treatment group in SKpac and SKOV3-inoculated mice. The combined treatment group with PTX + p110β siRNA in SKpac xenografts had a suppressed growth curve by 41, 68, and 63 %, respectively, when compared with groups with negative siRNA + PTX, or PTX alone and PBS only-treated groups. Points, mean; *bars*, SE; * *p* < 0.05 for PTX + p110β siRNA versus PTX alone and PBS only-treated groups

To further examine the in vivo effect of p110β silencing on ovarian cancer cell lines, female nude mice were inoculated s.c. with parent SKOV3 cells and chemoresistant SKpac cells. The appearance of the tumors after 35 days from the first day of treatment is shown in Fig. 4a. When mice were killed, and tumor tissues were separated and weighted, the tumor growth of p110β siRNA + PTX treatment groups in SKpac xenografts was obviously suppressed as compared to the control and PTX-only treated groups (Fig. 4b). The graphs depicting changed tumor volumes for SKpac and SKOV3 cells are presented in Fig. 4c, d. Tumors in SKpac-xenografted mice receiving the combined treatment with PTX + p110β siRNA had a suppressed growth curve by 41, 68, and 63 %, respectively, when compared with tumors in mice treated with negative siRNA + PTX, or PTX alone and PBS only-treated groups (*p* < 0.05, *t* test, Fig. 4c). Interestingly, the inhibitory effect of PI3K p110β siRNA on tumor growth was additive to the effect of PTX in mice inoculated with chemosensitive SKOV3 cells (*p* < 0.05, *t* test, Fig. 4d). Taken together, these in vivo results indicate that the growth of tumors was additively inhibited by p110β siRNA treatment both in SKOV3 and SKpac-xenografts.

### Apoptosis-related gene alteration associated with chemoresistance in ovarian cancer cells

**Fig. 5:**
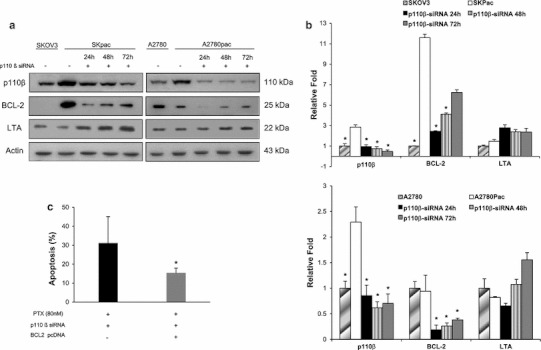
Protein expression of apoptosis-related genes significantly altered after PI3K p110β siRNA treatment in chemoresistant SKpac cells. **a** Protein expression of apoptosis-related genes significantly altered by PCR array in SKpac and A2780pac cells before and after PI3K p110β siRNA treatment. Actin was used as a loading control. **b** The graph represents the relative amount of each protein compared to their parent cells. Protein bands of (**a**) were quantitated by densitometric analysis. The intensity ratio to corresponding β-actin band was calculated. BCL 2 was overexpressed in SKpac cells compared with parent SKOV3 and A2780 cells, and was downregulated by 80, 70, and 40 % at 24, 48, and 72 h, respectively, after p110β siRNA treatment. LTA was increased after p110β knock-down. Representative experiment repeated twice with similar results. **c** The graph represents the mean apoptotic cell rates after combined treatment of PI3K p110β siRNA and PTX with or without pcDNA BCL2 transfection. Transfection of pcDNA BCL2 plasmid resulted in decreased apoptosis (15.44 ± 2.48 %) in SKpac cells treated with PI3K p110β siRNA and PTX, whereas the cells treated with PI3K p110β siRNA and PTX demonstrated increased apoptosis (30.02 ± 13.88 %) compared with PTX alone treated cells. Representative experiment repeated three times (* *p* < 0.05, *t* test)

To investigate the apoptosis-related genes induced by chemoresistance in ovarian cancer cells, we analyzed the gene expression profiles of parent SKOV3 cells and chemoresistant SKpac cells using a PCR array containing 84 key apoptosis genes. Based on gene selection criteria (*p* < 0.05 and ≥2-fold change), BCL-2 was up-regulated by 20-fold and 13 genes were down-regulated by ≥2-fold in chemoresistant Skpac cells, compared to parental SKOV3 cells (supplementary data Table S6). Next, to investigate for the effect of down-regulation of p110β, we further examined for the alteration of expression profile of apoptosis genes after transfection of p110β siRNA in SKpac cells. Eight genes including LTA (9.37-fold, *p* < 0.010), BCL 2 (−2.18-fold, *p* < 0.022) were significantly altered after p110β siRNA transfection (supplementary data Table S7). The alteration of these proteins was confirmed by Western blot; the alteration of BCL 2 and LTA correlated with the results of the PCR array. Intriguingly, BCL 2 protein was increased in chemoresistant SKpac cells by 521-fold compared with the parental SKOV3 cells, and was decreased by 78 % at 24 h after p110β siRNA transfection (Fig. 5a, b). LTA was not significantly altered in chemoresistant SKpac cells compared with the parental SKOV3 cells, but it was increased by 1.3–2-fold at 72 h after p110β siRNA transfection (Fig. 5a, b).

### BCL2 transfection decreases apoptosis induced by p110β siRNA

To assess whether the increased apoptosis by p110β silencing is caused by downregulation of BCL 2 in chemoresistant SKpac cells, TUNEL assay was performed after transfection of pcDNA3 BCL2 plasmid in p110β siRNA-treated SKpac cells. As expected, the increased apoptosis (30 %) by PI3K p110β siRNA treatment in SKpac cells was reduced to 15 % (*p* < 0.05, *t* test) at 48 h with treatment of pcDNA3 BCL2 plasmid (Fig. 5c).

## Discussion

PTX is an extensively used chemotherapeutic agent of various cancers including ovarian cancer. Resistance to PTX-based chemotherapy is a major cause of treatment failure in human ovarian cancer, which has the highest mortality rate among gynecological malignancies. Although the precise molecular mechanisms for the development of chemoresistance are poorly understood, accumulating evidence has indicated that the activation of the PI3K/Akt pathway is involved in the acquisition of resistance to chemotherapeutic agents in human cancers [6, 14]. PI3K/Akt is an important pathway in regulating many fundamental biological processes including cell proliferation, survival, apoptosis, glucose metabolism, and cell migration [19, 20]. Therefore, activated PI3K/Akt contributes to tumor development and progression. In addition, dysregulation of the PI3K/Akt pathway is also associated with the development of resistance to anti-cancer treatment. Aberrant PI3K/Akt activation is reportedly implicated in acquisition of chemoresistance [21, 22] as well as in the intrinsic chemosensitivity of malignancies. Therefore, inhibition of PI3K/Akt activation may be an attractive approach to treat human cancers and overcome the chemoresistance of cancer cells. However, nonspecific inhibition of broad-spectrum PI3K inhibitors may produce undesirable side effects because of the many important cellular targets of this lipid kinase, such as insulin regulation and immune response.

Class IA PI3Ks in mammals comprise three distinct catalytic subunits (p110α, p110β, and p110δ) according to their structure and interaction with p85 and p55 regulatory subunits [7]. These isoforms are considered to be different in their interaction with Ras and regulation of lipid kinase activity, and in their protein kinase activities [23, 24]. We show here for the first time that the p110β among the various isoforms of the PI3K p110 catalytic subunit is commonly overexpressed in a subset of ovarian serous carcinoma compared to the benign serous tumor, and that β-isoform overexpression is associated with PTX resistance in ovarian cancer cell lines. PI3K p110α also displayed a comparable increase in these carcinoma samples, however, it was not significantly increased in PTX-resistant cancer cells compared to chemosensitive cancer cells.

Among the various isoforms of Class IA PI3Ks, p110α and β are ubiquitously expressed in many human tissues and cell lines. The increased expression of the p110α isoform has been reported in many human cancers including ovarian, cervical and head and neck cancers [9, 25], and has been delineated for isoform-specific targeted therapy in many human cancers including ovarian cancer [26]. On the other hand, the p110β isoform has not been concerned compared with the p110α until a very recent study demonstrating kinase-dependent and -independent functions of PI3K p110β in cell growth and oncogenic transformation [27]. In the latter, ablation of p110β, but not p110α, impeded tumorigenesis with deminution of Akt-phosphorylation in an animal model of prostate tumor formation induced by PTEN knock-out. The same authors also reported that p110β had little effect on insulin signaling. Therefore, it was suggested that p110β may be an attractive target for kinase inhibitors in cancer treatment with minor metabolic disturbances.

In the present study, we demonstrate for the first time that specific inhibition of the PI3K p110β isoform, but not p110α, resensitizes chemoresistant ovarian cancer cells, suggesting its potential ability for the therapeutic target on chemoresistant ovarian cancers. Targeting p110β siRNA induced apoptosis and impaired cell proliferation in PTX-resistant SKpac cells, which showed no effect with PTX-only treatment. We also investigated whether the specific inhibition of p110α was able to resensitize chemoresistant ovarian cancer cells to PTX, because p110α is a major isoform for the signaling and growth of tumors driven by oncogenic RTKs/RAS [28], and targeting PI3K p110α was reported to inhibit tumor cell proliferation, chemoresistance, and migration in medulloblastoma cells [29]. Indeed, we observed its overexpression in serous carcinoma tissues and chemoresistant ovarian cancer sublines, however, the ablation of p110α did not lead to resensitization to PTX in the chemoresistant ovarian cancer cells, as shown in TUNEL assay and MTT cell viability assay, which demonstrated no increase of apoptotic cells and of cell viability, respectively, after p110α siRNA treatment.

The effect of selective PI3K p110β inhibition was also evident in vivo, in which the growth of tumors was additively inhibited by PI3K p110β siRNA treatment compared with negative siRNA + PTX, PTX-only treatment, and control groups, both in chemosensitive SKOV3 and chemoresistant SKpac xenografts. Previous studies demonstrating strong inhibition of cell growth and PI3K pathway signaling by targeted p110β down-regulation were restricted to PTEN-deficient cancer cells in both cell-based and in vivo settings [27]. Another study [30] observed that the down-regulation of p110α, but not p110β, resulted in PI3K pathway inactivation and cell growth inhibition in tumors with PIK3CA-activating mutation even with coexisted PTEN loss of function mutation. Thus, those authors suggested the p110β-targeted therapy particularly for PTEN-deficient cancers. Considering that SKOV3 and A2780 have a PIK3CA-activating mutation, however, our results provide strong evidence that p110β-targeted therapy could be useful for the treatment of chemoresistant cancer cells, even in the condition of PIK3CA-activating mutations, not only for the PTEN-deficient cancers.

Akt, the most important downstream effector of the PI3Ks, is activated by phosphorylation on two residues, T308 and S473. Regarding the western blotting for PI3K/Akt/mTOR signaling after p110β-specific inhibition, the function of PI3K p110β may be operated by Serine 473 Akt phosphorylation, not by Threonine 308 phosphorylation, which may be mediated by DNA-PK. The mTOR and phospho-mTOR, critical effectors of PI3K pathway, were sufficiently downregulated by p110β-specific inhibition. We also observed that cyclin E was clearly downregulated in PI3K p110β siRNA-treated SKOV3 cells, but cyclin D1 was not. This finding was not in accordance with the previous reports showing that inhibition of PI3K by LY-294002 greatly decreased the expression of cyclin D1 in ovarian cancer cells [31]. Taken together, PI3K may mediate G1-S progression by cyclin D1 induction, however p110β-isoform is unlikely to directly regulate cyclin D1. In addition, we found that cyclin-dependent kinase inhibitors, p21 and p27 were unexpectedly decreased by the treatment of PI3K p110β siRNA. Although some previous studies demonstrated the increased levels of p21 and p27 cyclin-dependent kinase inhibitors [32], several recent studies with ovarian cancer cells [31, 33] reported the decreased expression of p21 and p27 by PI3K inhibitor LY-294002 treatment, which were consistent with our results. These data indicate that p21 and p27 may not be relevant for cell proliferation regulated by PI3K in ovarian cancer cells. S-phase kinase protein 2 (SKP2), an F-box protein, targets cell cycle regulators, and is frequently overexpressed in a variety of cancers, including ovarian cancer. SKP2 is reportedly controlled at the transcriptional level by PI3K signaling [34]. Our result showing the marked decrease of SKP2 protein after PI3K p110β-siRNA treatment confirmed that SKP2 is a downstream target of PI3K p110β isoform in ovarian cancer cells. It has been known that Akt/PKB activate I-κB kinases that regulate the activity of the NFκB transcription factor, which is involved in multiple cellular processes including cell cycle activation and apoptosis [35]. We, thus, examined the protein level of NFκB, and found that NFκB was obviously decreased by p110β knock-down, implying that NFκB is a target PI3K p110β isoform.

Intriguingly, restoration of apoptosis after treatment of p110β inhibition seemed to be mainly caused by down-regulation of BCL 2, which mRNA and protein were elevated by the highest fold (20- and 591-fold, respectively) among the apoptosis-related genes in chemoresistant SKpac cells compared with chemosensitive SKOV3 cells. We confirmed this hypothesis by showing that transfection of BCL2 significantly decreased apoptosis induced by p110β-siRNA treatment in SKpac cells. Therefore, it can be explained that PI3K-p110β/Akt/mTOR pathway controls transcriptional activation of the BCL-2 anti-apoptotic gene, which are closely related with chemoresistance.

Lymphotoxin α (LTA) is a pleiotropic cytokine which mediates a large variety of inflammatory response and plays a role in the evolution and treatment of malignant disease [36]. LTA is considered as an anti-tumor factor inducing apoptosis through the death receptor p55 TNFR and the NFκB pathway [37]. With respect to our results showing the increase of LTA after down-regulation of PI3K p110β, LTA might be involved in PI3K p110β -induced apoptotic pathway.

In summary, our results presented in this study show that the specific inhibition of PI3K isoform p110β, but not p110α, is able to resensitize PTX-resistant cancer cells to PTX in vitro and in vivo, and represents a novel therapeutic strategy for overcoming the PTX-resistance in ovarian cancer. We also demonstrated that Bcl-2, cyclin E, SKP2 and NFκB are involved in this process.

## Electronic supplementary material

Below is the link to the electronic supplementary material.

Supplementary material 1 (PPT 164 kb)

Supplementary material 2 (DOCX 27 kb)
