# Supplementary material for: Targeted inhibition of phosphatidyl inositol-3-kinase p110β, but not p110α, enhances apoptosis and sensitivity to paclitaxel in chemoresistant ovarian cancers
Source: Apoptosis. 2013 Jan 31;18(4):509–20. doi: 10.1007/s10495-013-0807-9 (PMC3604599; doi:10.1007/s10495-013-0807-9)
Supplement: Supplementary file 1 — Supplementary material 1 (PPT 164 kb) [file 10495_2013_807_MOESM1_ESM.ppt]

## Slide 1
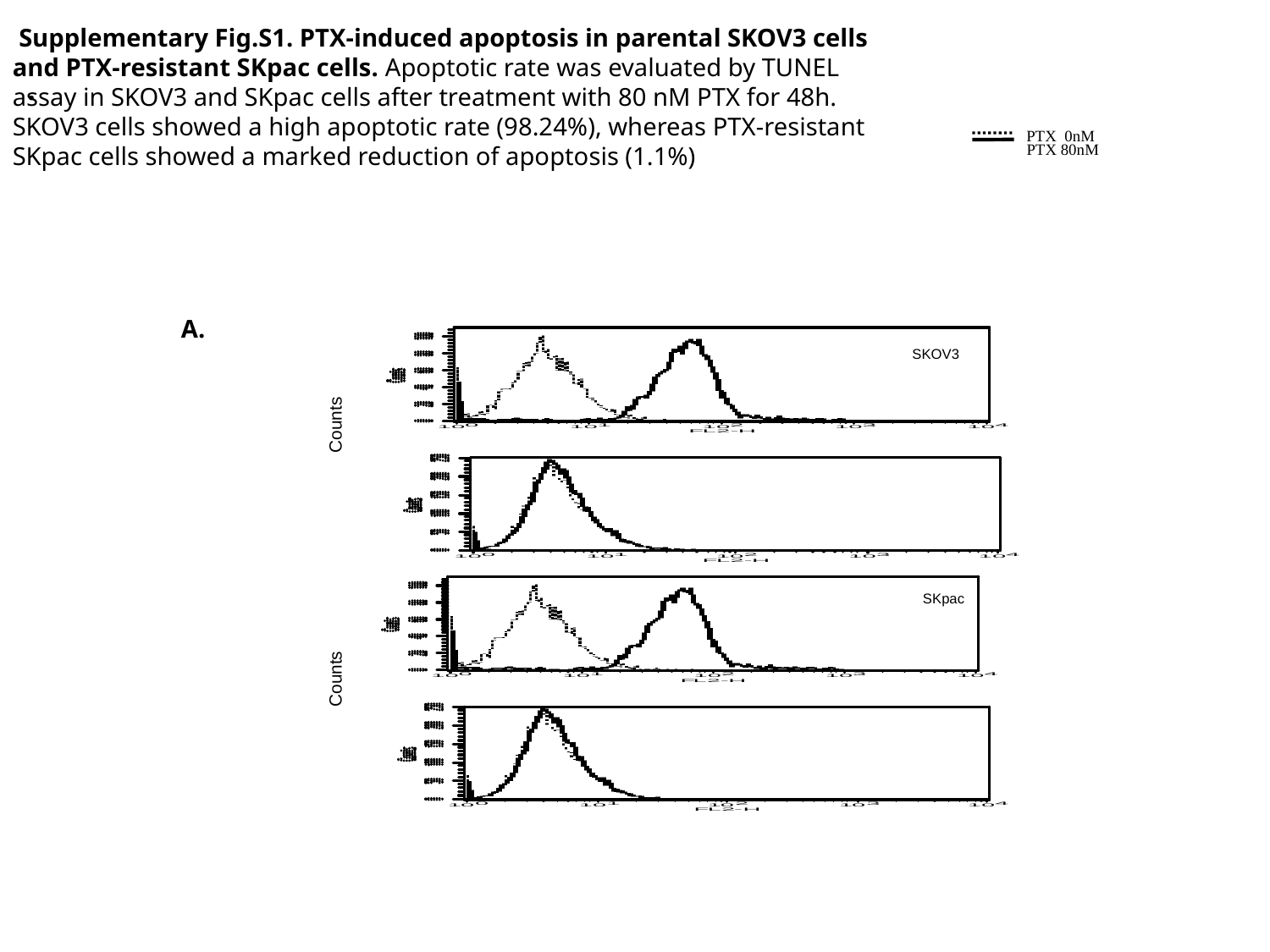

Supplementary Fig.S1. PTX-induced apoptosis in parental SKOV3 cells and PTX-resistant SKpac cells. Apoptotic rate was evaluated by TUNEL assay in SKOV3 and SKpac cells after treatment with 80 nM PTX for 48h. SKOV3 cells showed a high apoptotic rate (98.24%), whereas PTX-resistant SKpac cells showed a marked reduction of apoptosis (1.1%)
.
PTX 0nM
PTX 80nM
A.
SKOV3
Counts
SKpac
Counts

## Slide 2
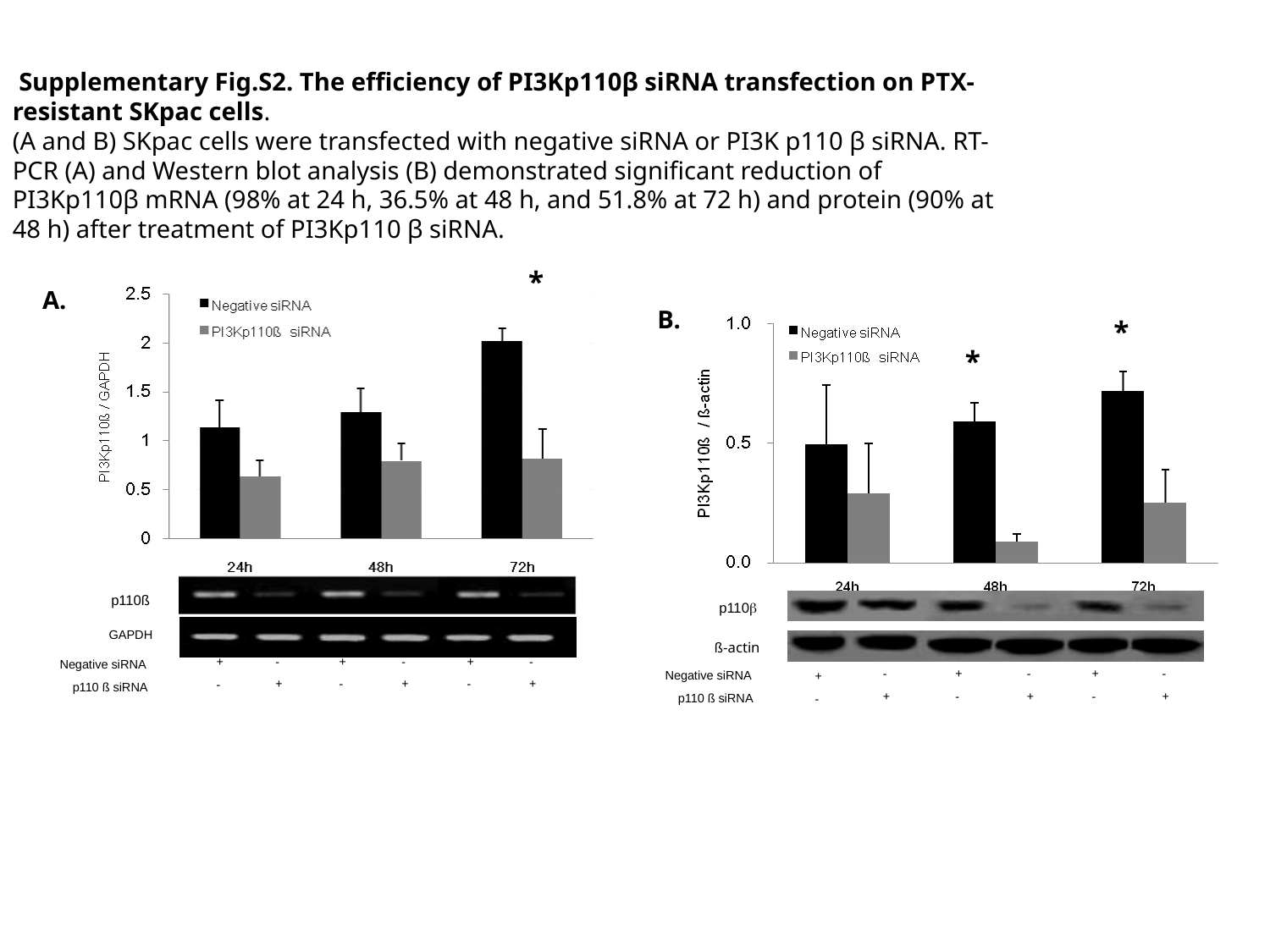

Supplementary Fig.S2. The efficiency of PI3Kp110β siRNA transfection on PTX-resistant SKpac cells.
(A and B) SKpac cells were transfected with negative siRNA or PI3K p110 β siRNA. RT-PCR (A) and Western blot analysis (B) demonstrated significant reduction of PI3Kp110β mRNA (98% at 24 h, 36.5% at 48 h, and 51.8% at 72 h) and protein (90% at 48 h) after treatment of PI3Kp110 β siRNA.
*
A.
 p110ß
GAPDH
-
+
+
-
-
+
+
-
-
+
+
-
 Negative siRNA
 p110 ß siRNA
B.
 p110β
ß-actin
 Negative siRNA
 p110 ß siRNA
-
+
+
-
-
+
+
-
-
+
+
-
*
*
