# Supplementary material for: Targeted inhibition of phosphatidyl inositol-3-kinase p110β, but not p110α, enhances apoptosis and sensitivity to paclitaxel in chemoresistant ovarian cancers
Source: Apoptosis. 2013 Jan 31;18(4):509–20. doi: 10.1007/s10495-013-0807-9 (PMC3604599; doi:10.1007/s10495-013-0807-9)
Supplement: Supplementary file 2 — Supplementary material 2 (DOCX 27 kb) [file 10495_2013_807_MOESM2_ESM.docx]

**Supplementary Tables**

**Table S1.** The sequences of PI3K p110α and p110β, and their siRNA oligos

| **Target gene** | **Sequences** |
| --- | --- |
| PI3K p110α | CTCCGTGAGGCTACATTAATA |
| PI3K p110β | CCCTTCGATAAGATTATTGAA |
| **SiRNA Oligos** |  |
| p110α - sense | 5’-CGUGAGGCUACAUUAAUATT-3’ |
| p110α - antisense | 5’-UAUUAAUGUAGCCUCACGGAG-3’ |
| p110β - sense | 5’-CUUCGAUAAGAUUAUUGAATT-3’ |
| p110β - antisense | 5’-UUCAAUAAUCUUAUCGAAGGG-3’ |

**Table S2.** Cell viability and IC50 of SKOV3 cells assessed by MTT assay with treatment of various concentration of PTX

| \| **PTX(μM)** \| **Viability(%)** \| \| --- \| --- \| \| **0** \| 100.00 \| \| **0.0003** \| 90.74 \| \| **0.0006** \| 108.77 \| \| **0.00125** \| 101.91 \| \| **0.0025** \| 94.62 \| \| **0.005** \| 90.77 \| \| **0.01** \| 69.09 \| \| **0.02** \| 55.47 \| \| **0.04** \| 40.74 \| \|  \|  \| \| **IC50** \| 0.022μM \| |  |
| --- | --- | --- | --- | --- | --- | --- | --- | --- | --- | --- | --- | --- | --- | --- | --- | --- | --- | --- | --- | --- | --- | --- | --- | --- | --- |

**Table S3.** Cell viability and IC50 of SKpac cells assessed by MTT assay with treatment of various concentration of PTX

|  |  |  |  | **Viability(%)** | |  |  |
| --- | --- | --- | --- | --- | --- | --- | --- |
| **PTX(μM)** | **SKpac8** | **SKpac10** | **SKpac11** | **SKpac12** | **SKpac13** | **SKpac16** | **SKpac17** |
| **0** | 100.0 | 100 | 100 | 100 | 100 | 100 | 100 |
| **0.06** | 92.1 | 92.7 | 89.9 | 89.7 | 86.4 | 94.5 | 103.8 |
| **0.08** | 94.3 | 92.5 | 87.2 | 101.6 | 85.7 | 92 | 94.3 |
| **0.12** | 95.3 | 89.7 | 80.8 | 96.8 | 84.3 | 90.9 | 90.3 |
| **0.16** | 91.6 | 88.2 | 73 | 93.9 | 86.8 | 91.1 | 92.7 |
| **0.24** | 78.7 | 81.7 | 77.4 | 91.4 | 79.4 | 82.2 | 81.2 |
| **0.32** | 86.0 | 79.8 | 69.9 | 81.4 | 83.3 | 83.6 | 74.3 |
| **0.48** | 71.0 | 68.3 | 58.4 | 68 | 83.3 | 72.7 | 56.5 |
| **0.64** | 80.8 | 69.8 | 58.6 | 68.3 | 80.7 | 75.9 | 54.2 |
| **0.96** | 73.7 | 63.8 | 64.2 | 61.4 | 76.3 | 61.8 | 65.5 |
| **1.28** | 75.2 | 64.6 | 61.6 | 67 | 73.2 | 63.6 | 57 |
| **1.92** | 74.5 | 62.5 | 62.4 | 61.6 | 70.4 | 58.1 | 58.1 |
| **2.56** | 70.6 | 60.8 | 58.4 | 58 | 68.8 | 60.1 | 58.9 |
| **3.84** | 64.3 | 58.1 | 55.9 | 57.5 | 64.7 | 57.3 | 50.9 |
| **5.12** | 61.1 | 56.5 | 52.7 | 58.5 | 63.3 | 54.8 | 50.4 |
| **7.68** | 53.8 | 49.8 | 48.5 | 43.6 | 54.6 | 52.8 | 45.4 |
| **10.24** | 50.1 | 48.3 | 47.1 | 42.1 | 43 | 42.6 | 44.7 |
| **15.36** | 23.9 | 23.3 | 18.4 | 14.9 | 27.1 | 27.7 | 28 |
| **20.48** | 22.0 | 21.6 | 16.8 | 13.8 | 26.6 | 24.4 | 26.2 |
| **30.72** | 4.7 | 5.7 | 4.3 | 4.6 | 12.2 | 4.4 | 4.1 |
|  |  |  |  |  |  |  |  |
| **IC50** | 10.21μM | 7.59μM | 6.77μM | 6.57μM | 8.69μM | 8.38μM | 5.32μM |

**Table S4.** Cell viability and IC50 of A2780 cells assessed by MTT assay with treatment of various concentration of PTX

| **PTX(nM)** | **Viability(%)** |
| --- | --- |
| **0.625** | 100.00 |
| **0.75** | 73.66 |
| **1.25** | 72.47 |
| **1.5** | 70.95 |
| **2.5** | 65.17 |
| **3** | 66.31 |
| **5** | 52.88 |
| **6** | 47.66 |
| **10** | 37.94 |
| **12** | 43.1 |
| **20** | 25.1 |
|  |  |
| **IC50** | 5.4nM |

**Table S5.** Cell viability and IC50 of A2780pac cells assessed by MTT assay with treatment of various concentration of PTX

| **PTX(nM)** | **Viability(%)** |
| --- | --- |
| **0** | 100.00 |
| **27.5** | 98.61 |
| **55** | 96.48 |
| **110** | 75.34 |
| **230** | 62.85 |
| **470** | 46.33 |
| **930** | 39.12 |
| **1875** | 26.85 |
|  |  |
| **IC50** | 430nM |

**Table S6.** Apoptosis related genes significantly altered in SKpac cells compared to parental SKOV3

| **Gene** | **Fold** **p-value** | | **Gene description** |
| --- | --- | --- | --- |
| BCL2 | 20.84 | 0.00 | B-cell CLL/lymphoma 2 |
| TNFRSF10A | -6.19 | 0.00 | Tumor necrosis factor receptor superfamily, member 10 |
| BIK | -5.32 | 0.00 | BCL2-interacting killer (apoptosis-inducing) |
| TNF | -4.29 | 0.02 | Tumor necrosis factor (TNF superfamily, member 2) |
| TP53 | -4.15 | 0.00 | Tumor protein p53 |
| TNFRSF9 | -3.80 | 0.02 | Tumor necrosis factor receptor superfamily, member |
| GADD45A | -3.38 | 0.00 | Growth arrest and DNA-damage-inducible, alpha |
| TNFRSF25 | -3.00 | 0.00 | Tumor necrosis factor receptor superfamily, member 25 |
| TRAF4 | -2.99 | 0.00 | TNF receptor-associated factor4 |
| TRAF3 | -2.78 | 0.00 | TNF receptor-associated factor3 |
| TNFRSF10B | -2.62 | 0.00 | Tumor necrosis factor receptor superfamily, member 10 |
| BAG4 | -2.13 | 0.01 | BCL2-associated athanogene 4 |
| CARD6 | -2.08 | 0.05 | Caspase recruitment domain family, member 6 |
| CASP8 | -2.06 | 0.01 | Caspase 8, apoptosis-related cysteine peptidase |
| AKT1 | -2.04 | 0.01 | V-akt murine thymoma viral oncogene homolog 1 |

Fold ≥2 and p-value< 0.05 were considered statistically significant.

**Table S7.** Apoptosis related genes significantly altered in SKpac cells after transfection of PI3Kp110β

siRNA.

| **Gene** | | **Fold** **p-value** | | | | **Gene description** |
| --- | --- | --- | --- | --- | --- | --- |
| TNFRSF11B | 109.33 | | | 0.030 | Tumor necrosis factor receptor superfamily, member 11 | |
| LTA | 9.37 | | | 0.015 | Lymphotoxin alpha (TNF superfamily, member 1) | |
| HRK | 2.67 | | | 0.041 | Harakiri, BCL2 interacting protein | |
| BFAR | 2.39 | | | 0.016 | Bifunctional apoptosis regulator | |
| BAK1 | 2.20 | | | 0.036 | BCL2-antagonist/killer 1 | |
| CARD6 | 2.15 | | | 0.01 | Caspase recruitment domain family, member 6 | |
| BAX | 2.00 | | | 0.049 | BCL2-associated X protein | |
| BCL2 | -2.06 | | 0.029 | | B-cell CLL/lymphoma 2 | |

Fold ≥2 and p-value< 0.05 were considered statistically significant.
